# The Immunoregulatory Potential of Particle Radiation in Cancer Therapy

**DOI:** 10.3389/fimmu.2017.00099

**Published:** 2017-02-06

**Authors:** Daniel K. Ebner, Walter Tinganelli, Alexander Helm, Alessandra Bisio, Shigeru Yamada, Tadashi Kamada, Takashi Shimokawa, Marco Durante

**Affiliations:** ^1^Hospital of the National Institute of Radiological Sciences, National Institutes of Quantum and Radiological Science and Technology, Chiba, Japan; ^2^Brown University Alpert Medical School, Providence, RI, USA; ^3^Trento Institute for Fundamental Physics and Applications-National Institute for Nuclear Physics (TIFPA-INFN), University of Trento, Trentino, Italy; ^4^Center for Integrative Biology CIBIO, University of Trento, Povo, Trentino, Italy; ^5^National Institute of Radiological Sciences, National Institutes of Quantum and Radiological Science and Technology, Chiba, Japan; ^6^Department of Physics, University Federico II, Naples, Italy

**Keywords:** immunotherapy, particle therapy, proton, carbon, abscopal

## Abstract

Cancer treatment, today, consists of surgery, chemotherapy, radiation, and most recently immunotherapy. Combination immunotherapy-radiotherapy (CIR) has experienced a surge in public attention due to numerous clinical publications outlining the reduction or elimination of metastatic disease, following treatment with specifically ipilimumab and radiotherapy. The mechanism behind CIR, however, remains unclear, though it is hypothesized that radiation transforms the tumor into an *in situ* vaccine which immunotherapy modulates into a larger immune response. To date, the majority of attention has focused on rotating out immunotherapeutics with conventional radiation; however, the unique biological and physical benefits of particle irradiation may prove superior in generation of systemic effect. Here, we review recent advances in CIR, with a particular focus on the usage of charged particles to induce or enhance response to cancerous disease.

## Introduction

The traditional approach to cancer treatment has primarily consisted of three central modalities: surgery, radiation, and chemotherapy, the first two indicated for management of gross, macroscopic disease and the latter to target microscopic and systemic disease. Advances in biomolecular understanding of cancer has lead to enhanced focus on the role of the immune system in clearing disease, and today, modulation and enhancement of the immune system, immunotherapy, has emerged as the fourth pillar of cancer management.

Combination immunotherapy–radiotherapy (CIR) experienced a surge in public attention with publication of numerous clinical accounts of metastatic disease remission following combination treatment with radiotherapy and ipilimumab, a cytotoxic T-lymphocyte antigen 4 (CTLA-4) inhibitor (1–3). Preclinical and clinical investigations exploded soon thereafter; a search for “radiation + immunotherapy” on http://ClinicalTrials.gov in December 2016 yielded 323 results. The mechanism behind CIR remains unclear, though consensus may be building for radiation potentiating an immune response to a tumor, forming an *in situ* vaccine that, with proper immune checkpoint modulation, can amplify the immune response systemically through blood and lymph, overcoming tumor microenvironment immunosuppression (4). As such, CIR is increasingly considered one of the most promising strategies to defeat cancer.

Here, we review recent advances in CIR, with a particular focus on the usage of charged particles to induce or enhance response to cancerous disease.

## Radiotherapy

Though innumerable immunotherapeutics are in testing, radiation therapy worldwide has largely used a single type, X-ray irradiation. This consists of an external beam of radiation delivered directly to target tumor tissue, producing a generally uniform dose that decreases slightly from body entrance to exit, irradiating target tumor and healthy tissue equivalently. To avoid unnecessary healthy-tissue damage, dose is delivered in multiple fractions, with healthy tissue self-repairing while DNA damage accumulates in the generally repair-deficient tumor. Further, the beam is often delivered from multiple angles or in an arc, collating dose in the tumor while minimizing total radiation exposure to healthy tissue. Conventional X-radiotherapy operates under a twofold mechanism. The first involves direct DNA damage, with energy delivered causing single-strand breaks and occasionally double-strand breaks in DNA; if the cell is unable to repair, it will undergo apoptosis or necrosis. Second, radiation has an indirect effect through creation of oxygen free-radicals in the target beam path, which lead to further local damage. The principle challenge of radiotherapy thus hinges on the inherent radioresistence of target tissue in relation to the radiosensitivity of surrounding normal tissue, and so the ability to deliver maximal target dose with minimal surrounding is paramount.

**Figure 1:**
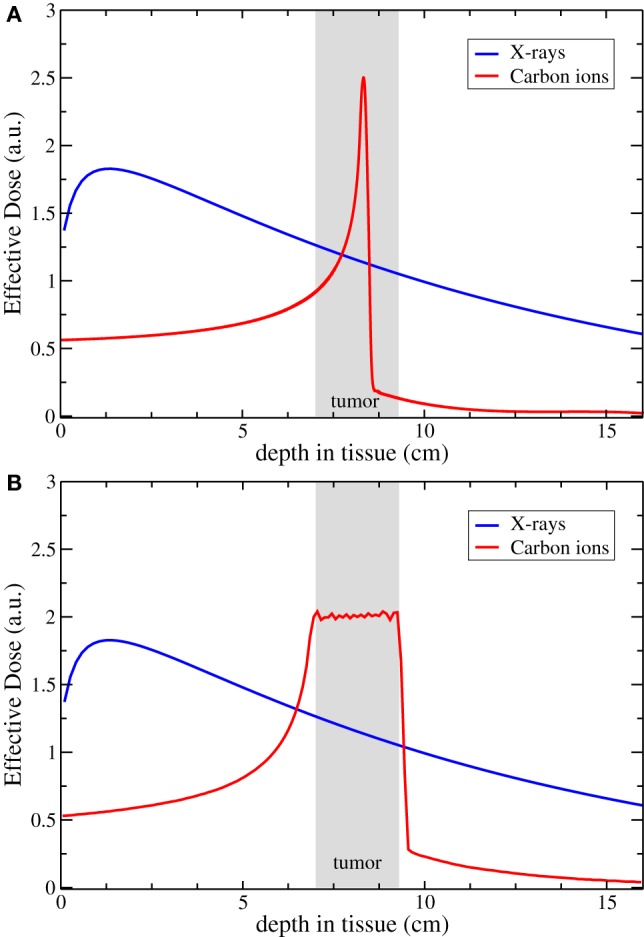
**Comparison of the dose distribution for carbon ion and X-rays**. Panel **(A)** shows the physical profile of a single peak compared to a typical photon irradiation; in panel **(B)**, the resulting profile of a biologically effective dose obtained with a Spread-Out Bragg Peak. Courtesy of Dr. Scifoni, Trento Institute for Fundamental Physics and Applications (TIFPA-INFN).

Particle radiotherapy (PRT) has been in various stages of research and development for 70 years, and today, clinical treatment is available in the form of either proton or carbon-ion radiotherapy. PRT operates by accelerating single particles to high velocities and directing them toward target tissue, with distance traveled in tissue a function of particle energy. As the particle slows, the number of ionization events with its surrounding environment increases, resulting in a dose-release spike known as the Bragg Peak (Figure 1A). This results in a comparatively low entry dose and little-to-no exit dose compared with X-ray irradiation. Smaller particles, such as proton, have a sharper distal dose edge but generate a slight penumbra due to scattering in tissue; heavier ions have a slightly higher exit dose due to nuclear fragmentations, with sharper lateral margins. To deliver target dose to the entire body of the tumor, the Bragg peaks are overlapped to form a spread-out Bragg peak (Figure 1B).

Originally, this was performed using a series of collimators and range filters to spread the beam, generating an excess neutron dose to the overall body of the target. However, recent advances allowed first proton and now heavy-ion beams to be actively scanned point-by-point across the target, eliminating excess dose and allowing improved dose delivery (5).

In addition to the dose-distributive benefits afforded by particle beams, heavy-ion beams have a high linear energy transfer (LET), that is, a higher amount of energy per particle transferred per unit distance. This increased number of ionization events delivered in a shorter distance interval yields an enhanced probability for double strand DNA breaks among other effects within a tumor cell; this is related to the biological damage delivered per unit dose by calculated comparison to an equivalent photon dose, and is termed the relative biological effectiveness (RBE). Original research in this area consisted of usage of neutron irradiation from the 1950s to 1970s, which demonstrated high LET but poor dose distribution; this lead to employment of proton, which offered superior dose distribution but little LET benefit. Principally from the 1990s, carbon-ions have been employed in Japan and Germany, offering both dose-distributive and LET benefits (6). The combination of dose-distribution benefit with an enhanced RBE 2 to 3× that of photon has lead to evidence that carbon-ions, owing to the direct DNA damage mechanism they employ, are relatively cell-cycle and oxygenation independent, and can be used to treat hypoxic and radioresistant disease (7). As the LET value of the carbon-ion and other heavy-ion beams varies throughout the beam path, future developments may involve “painting” high-LET values to target areas, further enhancing the biological effect (7, 8).

To date, radiotherapy has been thought of as a predominantly local treatment, with no systemic effect. However, X-irradiation has demonstrated involvement in both immunostimulation and immunosuppression (9, 10). Preclinical work has revealed that PRT appears to induce an identical or broader immunogenic response versus X-irradiation (11, 12), as well as evidence that carbon-ion beams induce anti-metastatic and anti-angiogenic effects.

## Cancer Immunosurveillance

In addition to the direct effects of radiation, surgery, and chemotherapy, the immune system plays a distinct role in recognizing and destroying cancer cells, as well as in clearing and repairing the damage caused by the first three methods. Burdet and Thomas first hypothesized that the immune system can recognize and eliminate transformed cells when they first occur, thus strongly decreasing cancer incidence than may be seen in immuno-incompetent individuals; though this hypothesis was abandoned, today evidence clearly supports it (13). Immunodeficient HIV patients have a noted increase in cancer incidence (14); cancer incidence appears to return toward baseline with fast administration of therapy (15). Choy et al. suggested that highly active antiretroviral therapy (HAART) improved glioblastoma survival in HIV+ patients, suggesting that HAART-enabled repopulation of the immune system’s white blood cell population improved outcomes, even in glioblastoma, which is largely protected from immune system interaction due to the blood–brain barrier (16).

This initial immunosurveillance hypothesis has come to be embodied by the “three E’s” of cancer development. The first is elimination. Cancer cells present new surface antigens that are recognized by the immune system as exogenous or “not-self,” leading to cancer cell recognition by antigen-presenting cells (APCs), immune activation, and facilitation of cancer cell elimination by CD8^+^ cytotoxic T-lymphocytes (CTLs). This generates a microevolutionary pressure in which only those cells able to avoid non-self antigen presentation, or to suppress the immune response in their environment, survive. The immune system thus shapes tumor progression, and this process is termed immunoediting (17). Avoidance of self-reporting to CTLs involves downregulation or inactivation of major histocompatibility complex I (MHC-I) antigen processing and presentation. Tolerogenic factors are further released by the cell to diminish surrounding CTL activity, modifying intratumoral dendritic cells (DCs), and recruiting and enhancing activity of regulatory DCs and T-cells (Treg), in addition to myeloid-derived suppressor cells (MDSCs) and tumor-associated macrophages. The result is an environment of diminished T-cell activity. This point is termed equilibrium, and occurs when elimination and replication of cancer cells is held in check, with the tumor unable to expand freely; the cells further accumulate mutations to enhance growth, immunosuppress the local environment, and to achieve metastatic potential. Finally, escape occurs, in which the growth of the cancer outpaces, through suppression and evasion, the immune system (18).

Treg cells may otherwise be termed suppressor T-cells, and are a subpopulation that serve to maintain self-tolerance and prevent autoimmune disease through suppression of active T-cells. They are formed in response to TGFβ expression, which further serves to maintain them. However, Treg immunosuppressive activity can be co-opted by tumors to contribute to their evolution toward escape; patients with a high Treg infiltration are known to have a poor prognosis (19). As these cells are considered to be contributory in the development of cancer, they form distinct targets in the tumor microenvironment. However, they are radioresistant, attenuating response and contributing to increased radioresistance following irradiation, while also increasing in number.

Myeloid-derived suppressor cells are further immunosuppressive, reducing the activation of other white cell populations. They serve to release the inflammatory cytokine prostaglandin E2 (PGE2), supporting tumor growth and cancer repopulation, while protecting tumor cells from apoptosis. PGE2 increases following irradiation, and its release is tied to the LET of the radiation employed, as well as oxygen concentration (20).

Radiation has been demonstrated as both immune-stimulating as well as immune-suppressive. This balance can be shifted using immunotherapeutics. Long-term clinical results, principally of melanoma remission following administration of radiotherapy and ipilimumab (3), have demonstrated the potential for this combination in a clinical setting. Subtotal responses reveal that further work is needed to overcome existing disease resistance, as well as to prevent disease adaptation to the blockade.

## Radiation and Immunoactivation

**Figure 2:**
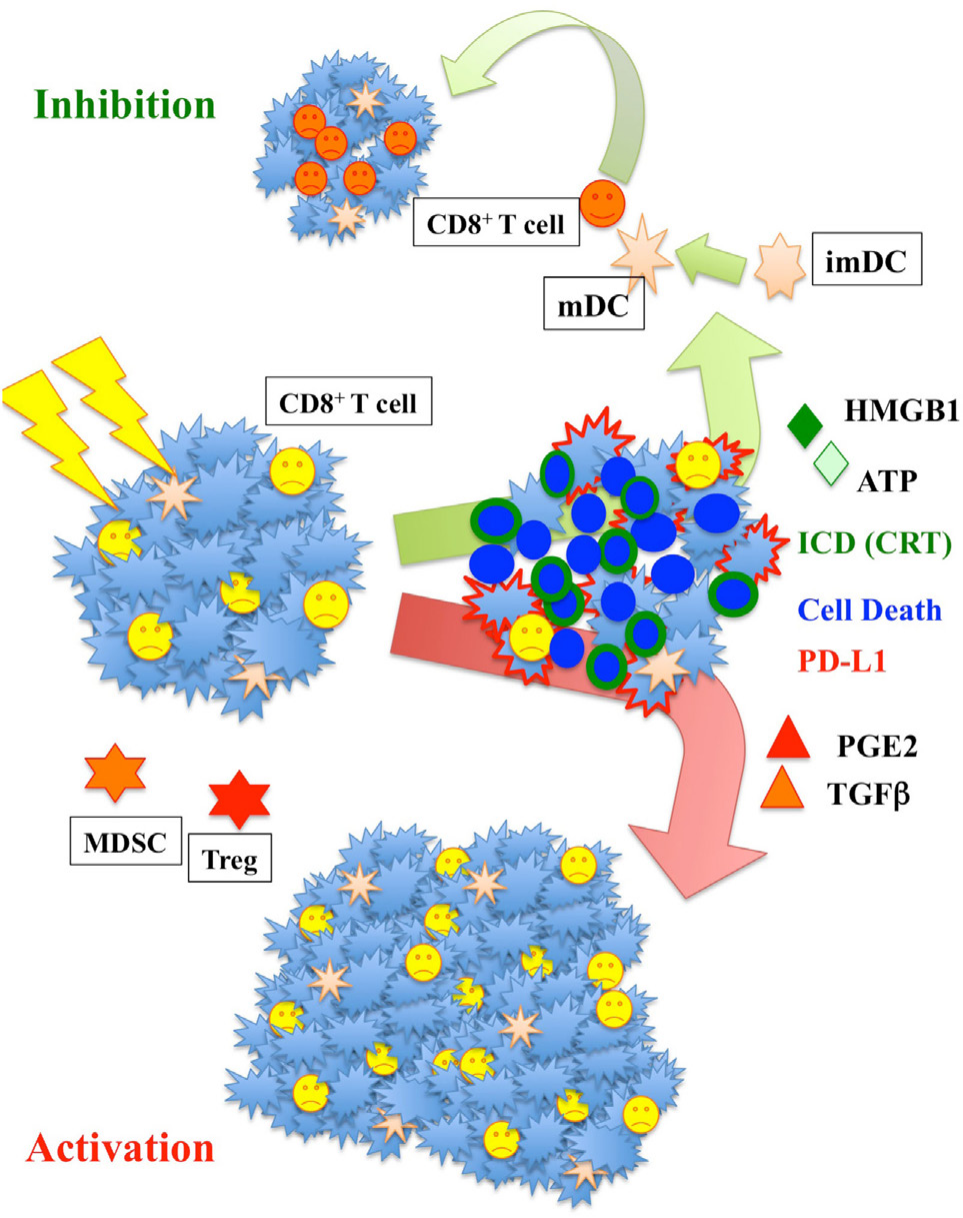
**Radiation increases major histocompatibility complex I and enhances calreticulin (CRT) translocation and ATP-high mobility group box-1 (HMGB1) release**. Those signals are fundamental to activate dendritic cells (DC). Activated DC increase their mobility and move to the lymph nodes where they activate T-cells. The increase of prostaglandin E2 (PGE2) and TGFβ could yet be counterproductive, increasing the population of myeloid-derived suppressor cells (MDSC) and Treg cells responsible for immunosuppression.

Radiation has a unique effect on tumor tissue, serving as a means by which to generate immunogenic cell death (ICD) within a tumor (Figure 2). Upon exposure to radiation, tumor cells present damage-associated molecular patterns (DAMPs), which enable the cell to be engulfed by APCs. These are in turn presented to CTLs, leading to tumor destruction. This pathway is facilitated by IRE1, PERK, and ATF6, which in low-stress conditions are inactivated by BiP/GRP78. Following irradiation, this inactivation diminishes, leading to DAMP trafficking to the surface. DAMP response to radiation appears to be dose dependent and varies significantly with tumor histology and genetics.

Within the DAMPs, calreticulin (CRT), ATP, high mobility group box-1 (HMGB1), and type I interferons appear to play the major role (21, 22). When a cancer cell is undergoing ICD, 6× normal concentration levels of CRT are found on the surface. This is dependent on PERK-mediated phosphorylation of EIF2α or inhibition of Eif2α-specific phosphatase complex PP1/GADD34. With radiation, this process appears to be mediated by Erp57. CRT interacts with SNAREs on the cell surface, interacts with the TNF family, and may activate complement C1q; essentially, it serves as a potent dendritic cell “eat me” signal. This signal may be counteracted by the antiphagocytic molecule CD47, considered the “do not eat me” signal (23), which is ubiquitously expressed in human cells and overexpressed in numerous tumors. Radiation seems to reduce the amount of CD47, increasing the rate of cell phagocytosis.

High mobility group box-1 is a highly conserved nuclear protein involved with replication, and is expressed in nearly all cells. It serves as an immunoactivating cytokine and DAMP when released by necrotic cells following ablative radiotherapy, activating DCs through toll-like receptor 4 (TLR4) (24). Inhibition of HMGB1–TLR4 interaction leads to earlier relapse in breast cancer patients, while HMGB1 levels following chemo-irradiation were predictive for survival, perhaps due to their effects on the proliferation of CTLs. Similarly, ATP and Interferon γ are released following irradiation in cells consequently undergoing ICD; ATP binds to P_2_Y_2_ and P_2_X_7_ purinergic receptor on macrophages and DCs, leading to activation of the DC inflammasome, secretion of IL-1β, and leading to inflammatory cytokine production. Meanwhile IFNγ increases following irradiation and enhances the level of APM-components, ultimately producing an increase of the MHC-I complex. Other mechanisms for immunoactivation secondary to radiation therapy include MHC-I activation *via* the CERAMIDE pathway, with VEGF-induced damage triggered by ASMase responsible for MHC-I enhancement (25). Less directly, blockage of type-I interferons or modification of TLR3 signaling stopped ICD in target tumors, suggesting a mechanism at work. Heat shock proteins 70 and 90 (Hsp70, Hsp90) are both directed to the cell surface during ICD, with released Hsp70 serving as a DAMP. Further, any DAMPs produced during ICD, regardless of radiation, also play a role in the downstream effects of ablative irradiation.

## Radiation and Immunosuppression

However, radiation is a two-sided coin, comprising not only immune activation but also suppression. The effect of radiation on the immune system itself is not well understood, with local dendritic and T-cells also being exposed to irradiation during treatment. Merrick et al. found that human myeloid DCs were very resistant to radiation-induced apoptosis and maintained their migratory and phagocytic capacities following radiation (26). However, irradiated DCs were less effective at activating lymphocytes and, when mature, were less able to produce immunoactivating IL-12 compared to control. As such, DC irradiation *in vitro* and, potentially, *in vivo*, may diminish immunoactivation and comparatively suppress immune response overall. This is in addition to any innate local immunosuppression caused by the naïve tumor; Merrick et al. thus suggested that irradiation of DC could shift the delicate balance from tumor regression to one of tumor expansion and escape. Patients exhibiting immunosuppression have been found to require higher radiation dose for local control (27).

More directly, upregulation of PD-L1 in tumor cells has been seen following irradiation, which Park and colleagues demonstrated can limit generation of an abscopal effect, in which distant disease regression is noted (28). Melanoma was injected into the hindlimbs of PD-1-deficient C57BL/6 mice and compared to wild-type following treatment of one limb with SABR; mice deficient in PD-1 saw a fivefold reduction in their untreated tumor. This suggests activity of PD-1 as an anti-abscopal marker. PD-1 operates by downregulating the immune system and promoting self-tolerance, inducing apoptosis in T-cells and overall contributing to generation of an immunosuppressive environment. Blockade of PD-1 allows systemic expansion of T-cells, increasing tumor infiltration. Consequently, blockade of PD-1 may allow for better triggering of the abscopal effect.

Further, radiation increases the amount of PGE2 cytokine (20), which not only contributes to the Phoenix Rising effect, in which surviving distant disease becomes more aggressive and fast growing following local treatment, but also increases the population of MDSCs and contributes to a shift of T-cells to Treg cells. This contributes to the immunosuppressive environment.

TGFβ is also released following irradiation, leading to immune suppression by increasing the ratio of Treg cells. It is unclear precisely what role TGFβ serves, though in mice it appears to be age-related. Co-activation with IL-6 produced by mature DCs appears to be an important parameter to shift TGFβ to immunoactivation over suppression (29). TGF-β is also implicated in B-lymphocyte proliferation and NF-κB inhibition. It increases the apoptosis of immature B-lymphocytes.

The decision to refrain from or to undergo ICD plays a major role in development of immunosuppression or immunoactivation. Following damage, cells may attempt self-repair or become apoptotic. Langerhans cells in the skin, following UV irradiation, are able to induce Treg cells and avoid immune self-destruction even if they are expressing foreign “non-self” antigens. This is thought to prevent autoimmune reactions in the skin, but the exact mechanism is unclear (30). However, this may be co-opted by tumors, facilitating escape from immune detection. Similar mechanisms exist: inhibition of macrophages, DCs, or T-cells can further be accomplished through the release of cytokines. IL-10, for instance, interferes with DC maturation by blocking T-cell activation.

## ICD and Radiotherapy

The irradiation and destruction of local immune system cells in theory may contribute to immunosuppressive shift. In this vein, particle irradiation may be beneficial due to a reduced integral dose and overall reduced irradiated volume compared with proton, limiting unnecessary destruction of lymphocytes (31, 32). Photon radiotherapy induces ICD in a dose-dependent manner *via* CRT translocation, and HMGB1 and ATP release (33), which are necessary for radiation treatment success (34). Depending in part on dose and type of radiation delivered, varying types of induced cell death may result: apoptosis, necrosis, mitotic catastrophe, necroptosis, or autophagy. When these processes occur with the translocation of CRT, HMGB1, and ATP release, or the dispersion/release/translocation of other immune-stimulating antigens from dying cells into the surrounding milieu, this leads to immune system activation and may be termed immunogenic cell death (35).

Traditionally, cell killing is ascribed to four basic principles, termed the “4 R’s of Radiobiology”: reassortment, reoxygenation, repair, and repopulation. Recent discussion has lead to the suggestion that ICD may be considered the fifth radiobiological principle, due to induction of immune system activation and potential generation of a systemic antitumor effect (36). Though the local consequences of irradiation in a tumor are readily apparent, with development of ICD due to direct irradiation effects, as well as induction of immune system response in the local environment, recent attention has turned to what role ICD may play in the generation of systemic effects. It is proposed that immunoactivation may extend beyond the local tumor, facilitating a system-wide antitumor response. Circulating levels of cytokines have been found following radiotherapy, with prostate adenocarcinoma patients and head and neck cancer patients both having detectable levels of inflammatory and/or fibrogenic factors in circulation following radiotherapy (37).

## Combination Immunotherapy and Radiotherapy

Usage of CIR to induce systemic regression of cancerous disease is hoped to revolutionize cancer treatment, allowing generation of bystander or abscopal effects, signifying regional or distant antitumor effect, respectively. Though the precise mechanism remains unknown, radiotherapy is thought to convert an individual tumor into an *in situ* vaccine, after which it serves as a way station for immune system activation, amplification, and proliferation in targeting systemic disease (34). This vaccination effect has been seen in colon cancer (38). The abscopal effect has been known for decades to occur with radiotherapy alone, although it was notably rare (39, 40). Clinical abscopal effects remained mechanistically elusive, until in 2004 when Demaria and colleagues suggested that it is immune-moderated (41). With the advent of modern immunotherapeutics, which can be administered to preserve, amplify, and regionosystemically expand these responses, the possibility of inducing a controlled abscopal effect is nearing reality. Mechanistically, the existence of radiotherapy-only abscopal effects suggest that the driving agent of the effect either occurs spontaneously or is secondary to radiation in a small proportion of patients, respectively. Immunotherapy thus aims to extend the potential for abscopal effect generation to a wider population.

Case reports have been seen in a variety of tumor histologies, though the most replicable thus far appear to focus on combination radiotherapy and CTLA-4 inhibition (*via* ipilimumab) in melanoma (1–3, 42). Melanomas (30–40%) have NY-ESO-1 and may thus be susceptible to ipilimumab. In one case, a patient was treated with ipilimumab and kept on maintenance. Palliative radiotherapy was applied to a paraspinal mass, with ipilimumab again delivered a month following. Two months thereafter, widespread disease regression was noted, with minimal stable disease 6 months later. Post-radiotherapy showed a 30-fold increase in antibodies against NY-ESO-1 protein (3). Addition of PD-1 blockade has yielded a similar abscopal effect against melanoma and RCC (37). A phase I trial of combination therapy in melanoma found increased PD-L1 expression following treatment, with less than 20% of patients developing abscopal-like reactions; blocking PD-L1 was suggested. DCs treated *ex vivo* with different activators and modifiers and then delivered intravascularly or intratumorly, in combination with radiation, have also demonstrated good results in multiple studies, and may be promising as a treatment amplification option. As DCs serve as the primary activators of the local immune response, direct DC injection is thought to improve the likelihood of overcoming environmental immunosuppressive effects.

Unfortunately, the precise mechanism behind clinical induction of CIR-mediated disease remission has yet to be understood and thus is difficult to replicate on a population basis, forming the central challenge behind clinical treatment with immunoradiotherapy. Due to the microevolutionary nature of cancer treatment and heterogeneity of tumors, any individual’s tumor ideally will be targeted with disease-, histology-, and perhaps genetic-level precision, as cells surviving initial treatment can expand unheeded. Mechanistic understanding of CIR is needed.

## Particle Combination Therapy

Particles have been theorized to increase the advantages and utility of CIR. Particles appear to demonstrate higher antitumor effects versus photon irradiation, with reports that they are more effective in reducing metastasis (43), while reducing or preventing local recurrence (44, 45).

The immunogenicity of radiation may correlate to the density of irradiation, with enhanced efficacy seen with high-LET, densely ionizing radiation in cell cultures (12, 46). Among other pathways, high LET radiation appears to increase the CERAMIDE pathway more efficiently than low LET X-ray (47). Across multiple tumor cell lines, protons mediated CRT translocation to the cell surface, increasing cross-priming and sensitivity to CTLs (11). This may be enhanced with more densely ionizing heavy ions, such as oxygen or carbon.

In mouse studies, in combination with DC injection, the carbon-ion beam correlated with a greater amount of immune activation (48). Though DC injection has been found promising with photon trials, as carbon-ions are generally used to treat deep-seated tumors, intratumoral dendritic cell injection may not be feasible with many carbon patients. Alternate delivery methods and/or alternate *in situ* DC amplification methods may be necessary (48, 49). Animal studies involving immunotherapeutic agents combined with carbon-ion irradiation are underway.

Carbon-ions have been linked to induction of an abscopal effect in combination with immunotherapy (48). There has been a theoretical response to carbon seen with pancreatic cancer patients, as well as abscopal-like effects seen with the carbon-ion beam. An 85-year-old patient received 50.4 Gy (RBE) in 12 fractions for an ascending colon carcinoma, with mediastinal lymph node metastases resolving 6 months following carbon-ion radiotherapy. Whether this is due to ablative dose delivery afforded by the carbon-beam, or an immunogenic effect secondary to the usage of high-LET radiation, remains to be elucidated.

## Unknowns and the Future

To this end, numerous avenues of radiotherapy and their effect on the systemic immune system remain to be clarified. Though combination reactions have been clinically demonstrated, they remain rare, with clinical trials commonly reporting maximal systemic disease regression rates of 20% or less. Tumors are now known to be heterogeneous, and so therapy that eliminates disease, and does not simply select for resistant disease, must be employed. Which combinations of immunotherapeutics are indicated in what diseases and histologies, the (epi)genetic profiles of those diseases, as well as variables such as whether surgery or chemotherapy are performed, timing and dose, as well as radiation usage, radiation type, dose, fractionation, and more, all may play a role in the delicate balance between immunoactivation and immunosuppression (50). Conventional fractionation regimens tend to settle at 2 Gy per fraction; hypofractionated fractions can deliver 20+ Gy per fraction, and appear to lead to greater immunoactivation. The reasoning for this may lie in the effect of fractionation on local lymphocytes: notably radiosensitive, lymphocytes invade the damaged tumor space, only to be repeatedly irradiated over the treatment period. Following classical irradiation protocols, the level of circulating lymphocytes in peripheral blood is notably low (51). Reduced fractionation may result in less peripheral lymphocyte death, and thus may serve to diminish systemic response in comparison with hypofractionation. Increasing dose would increase local CRT translocation, as well as HMGB1 and ATP release.

Nonetheless, *in vitro* studies suggest conventional fractionation is superior to hypofractionation in terms of activating the immune system. Rubner and colleagues found that fractionated radiation was better able to induce release of Hsp70, leading to DC maturation (52). Kulzer and colleagues similarly found that classical RT may better enable the tumor to serve as immunoactivation point, leading to a stronger immune response. They compared classical RT with single-dose protocols, finding elevated levels of immunoactivating cytokines IL-12p70, IL-8, IL-6, and TNF-α (53).

It has been demonstrated that different types of radiation are differentially efficient on differing tumor types, with some studies indicating cases where particle irradiation was less effective or equal to photon (54, 55). Modulation of dose and fractionation remain unclear: in an animal model, stereotactic ablative body radiotherapy was demonstrated to be superior to classic RT fractionation, while *in vitro* evaluation suggested that for immune activation, classical fractionation was superior. Tsai and colleagues found differences in gene expression between classical and single-dose protocols, with robust gene induction found in the fractionated protocol (56). These unknowns will require study in the future.

Technological availability is reaching the point where different tumor types can be targeted with different ions, depending on their suitability. Heavy-ion facilities are being built rapidly in the world, with 2 in Europe, 6+ in Asia, and plans to construct 2+ in North America. Switching from one ion to another takes only minutes, and preliminary evidence suggests unique benefits offered by proton (sharp distal dose), as well as carbon and oxygen (sharp lateral doses and high LET) (57). Helium and lithium may soon be employed; it is possible immunoactivation may respond differently with ion type, and so comprehensive studies of these combinations will be needed.

Combination immunotherapy and radiotherapy offers a powerful modality for the treatment of cancer, and for the first time in cancer treatment, a potential therapy resulting in total remission of stage IV, distant disease, may be mechanistically understood. Innumerable factors play a role: the specific targets of immunotherapeutics, *ex vivo* modulation and reimplantation of immunoactivating cells, surgery, chemotherapy, radiation, and the dose, type, and timing of all these treatments. It is hoped that with careful understanding of the mechanisms involved, for the first time the clinical view of distant, stage IV illness may be shifted from “palliative” to “curative.”

## Author Contributions

All authors listed have made substantial, direct, and intellectual contribution to the work and approved it for publication.

## Conflict of Interest Statement

The authors declare that the research was conducted in the absence of any commercial or financial relationships that could be construed as a potential conflict of interest.
